# *Sabethes* mosquitoes (Diptera: Culicidae) associated with bamboo internodes in Pariquera-Açu and Cananeia, São Paulo State, Brazil

**DOI:** 10.1590/0037-8682-0125-2025

**Published:** 2026-02-09

**Authors:** Rafael Oliveira-Christe, Antonio Ralph Medeiros-Sousa, Márcia Bicudo de Paula, Henrique de Freitas Benar, Jair Donizete da Silva, Luiz Carlos de Oliveira, Mauro Toledo Marrelli

**Affiliations:** 1 Universidade de São Paulo, Faculdade de Saúde Pública, Departamento de Epidemiologia, São Paulo, SP, Brasil.; 2 Universidade Federal do Rio Grande do Norte, Programa de Pós-Graduação em Biologia Parasitária, Departamento de Microbiologia e Parasitologia, Natal, RN, Brasil.

**Keywords:** Mosquitoes, Atlantic forest, Tree holes, Yellow fever, Ecology

## Abstract

**Background:**

Mosquitoes of the genus *Sabethes* are widespread in South and Central America, and some species are associated with yellow fever transmission. We aimed to investigate *Sabethes* fauna in bamboo internodes in remnants of the Atlantic Forest.

**Methods:**

Artificial holes were made in bamboo plants and monitored for the presence of immature mosquitoes for 12 months.

**Results:**

Ten species of the genus *Sabethes* were identified, including *Sa. aurescens, Sa. identicus, Sa. conditus, Sa. shannoni, Sa. albiprivus, Sa. purpureus, Sa. undosus, Sa.ignotus, Sa.soperi,* and *Sa. whitmani*.

**Conclusion:**

Artificial hole placement can increase the number of *Sabethes* species found in faunal investigations.

*Sabethes* Robineau-Desvoidy (Diptera: Culicidae) is a neotropical genus of mosquitoes found in wild environments where they develop in natural breeding sites of phytotelmata, such as tree holes and bamboo internodes^1^. Some species of this genus, including *Sa. chloropterus*, *Sa. Albiprivus,* and *Sa. cyaneus*, have been shown to be associated with circulation of the yellow fever virus^2^. Other species of this genus have been isolated from several arboviruses, such as those from Mayaro, Bunia, Ilheus and Saint Louis^1^.

The presence of *Sabethes* species in yellow fever transmission zones was documented in an earlier investigation^3^ of mosquito fauna. However, in ground-level surveys of Culicidae, the presence of *Sabethes* species is generally low because they are primarily canopy-dwelling^4^. Breeding site investigations have been shown to complement mosquito fauna surveys and this type of investigation is essential when studying *Sabethes* mosquitoes^5^. 

In the present study, we discuss the fauna of *Sabethes* mosquitoes collected from the remnants of the Atlantic Forest in two municipalities in São Paulo State, Brazil. We also describe a strategy in which we used holes that simulated those made by Lepidoptera and Coleoptera insects in bamboo (side holes made with an electric drill, 8 mm in diameter), thereby increasing the number of *Sabethes* individuals collected in the field. Bamboo internodes were explored and bamboo traps were located at 1-3 m. Immature *Sabethes* specimens were collected during fieldwork conducted in 2023 (June and November) and 2024 (January to October). For each period, collections were conducted once a month for a total of 12 field samples. Bamboo internodes (*Guadua* spp.) were examined at three sites. The vegetation in these localities is defined as ombrophilous, dense forest with a submontane profile (Pariquera-Açu) and lowlands (Cananeia) (Figure 1). One site is located in the municipality of Pariquera-Açu (site A, 24°44'43.2"S 47°51'02.9"W), whereas the other two are located in the Cananeia (site B, 24°51'59.2"S 47°52'42.2"W and site C, 24°28'22.4"S 47°32'39.3"W ). Water in bamboo internodes with small holes made by insects was investigated for the presence of *Sabethes* as a previous study has reported the presence of immature members of this genus at these breeding sites^6^. The distances of these collection sites to the core of urban areas are: Site A, 5.7 km distant from the urban core of Pariquera-Açu, and Sites B and C, which are 23 km and 35 km, respectively, distant from the urban core of Cananeia.

**FIGURE 1:**
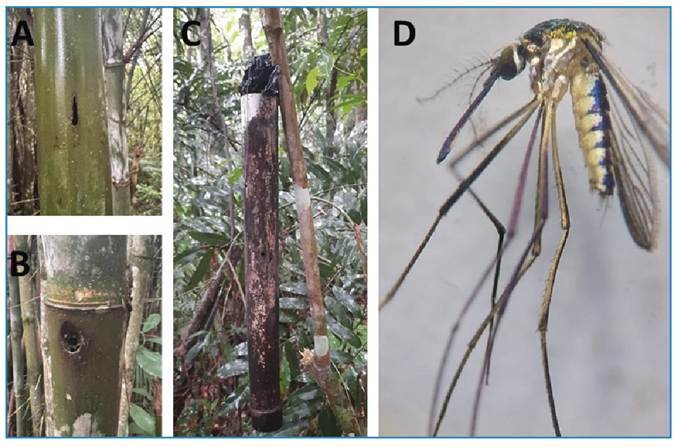
**(A)** Natural hole in bamboo made by Curculionidae species; **(B)** artificial hole made with an electric drill; C) bamboo trap with an artificial hole installed in site **(C)**; **(D)** specimen of *Sa. identicus* collected in bamboo internodes with artificial holes.

Although no bamboo plants were noted at site C, many adult individuals of *Sabethes* were collected using insect nets during exploratory field trips^3^. At Site A, the aquatic contents were sucked from the bamboo internodes with natural holes and artificial holes were made with an electric drill in some internodes. At Site B, the same procedure was executed. At Site C, four bamboo traps were installed because of the absence of natural bamboo. The artificial small holes resembled the natural holes made by Noctuidae larvae and adults of Curculionidae (Figure 1B). This procedure was used not to monitor and compare the efficiency of natural versus artificial holes, but rather to attempt to increase the number of immature insects collected. The larvae and pupae of *Sabethes* mosquitoes were collected using suction samplers. These specimens were then sent to the entomology laboratory at the School of Public Health, São Paulo University, and monitored until adults emerged, when they were identified using morphological keys^7^. The 10 different species of the genus *Sabethes* collected are listed in Table 1.

**TABLE 1:** Species of *Sabethes* collected in bamboo internodes in Cananeia and Pariquera-Açu.

Species	Site A	Site B	Site C	Total	Percentage %
*Sabethes* (*Peytonulus*) *aurescens* (Lutz), 1905	11	1		12	17.3
*Sabethes* (*Peytonulus*) *identicus* Dyar & Knab, 1907	8	2		10	14.4
*Sabethes* (*Sabethoides*) *conditus* Moses, Howard and Harbach, 2000			1	1	1.4
*Sabethes* (*Peytonulus*) *shannoni* (Lane & Cerqueira), 1942	2		2	4	5.7
*Sabethes* (*Sabethes*) *albiprivus* Theobald, 1903	7	3	8	18	26
*Sabethes* (*Sabethes*) *purpureus* (Theobald), 1907			5	5	7.2
*Sabethes* (*Peytonulus*) *undosus* (Coquillett), 1905	8	2		10	14.4
*Sabethes* (*Peytonulus*) *ignotus* Harbach, 1995	2	1		3	4.3
*Sabethes* (*Peytonulus*) *soperi* Lane & Cerqueira, 1942	2			2	2.8
*Sabethes* (*Peytonulus*) *whitmani* Lane & Cerqueira, 1942	3	1		4	5.7
**Total**	**43**	**10**	**16**	**69**	**100**

The most abundant species collected was *Sa. albiprivus* (18 individuals), followed by *Sa. aurescens* (12 individuals). While Site A exhibited the greatest abundance and richness of *Sabethes* species, Site C had the lowest values for both parameters. *Sa. albiprivus* was the only species found at all three localities. *Sa. conditus* and *Sa. purpureus* were only collected at Site C, whereas *Sa. soperi* was collected only at Site A. 

The holometabolic cycle of mosquito development depends entirely on water bodies for larval development. These bodies can vary in size, water volume, biotic communities and physicochemical composition^1^. The finding of immature *Sabethes* in bamboo internodes in previous studies can be attributed to the fact that such habitats provide suitable physicochemical conditions and microorganisms that serve as nutrients for the larvae of these mosquitoes^8^. 

Some *Sabethes* species such as *Sa. albiprivus* and *Sa. purpureus* are generalists and can be found in bamboo internodes and tree holes^9^, whereas others, such as *Sa. aurescens,* are more specialized and are found only in bamboo internodes^10^. The differences in abundance and diversity between the Sites suggest ecological differences between *Sabethes* species. Site C has primary Atlantic Forest flora with a greater availability of potential breeding sites (tree holes), Site B is in a human-impacted area with a highway running alongside it, and Site A is an area with transitional forest cover. The finding of a greater number of species and specimens at Site A indicates the capacity of species of this genus to adapt. 

The high number of *Sabethes* species collected in this survey indicates plasticity in the oviposition site selection behavior of some of these species. *Sa. albiprivus* and *Sa. purpureus,* which are typically found in tree holes and bamboo internodes, was found only in bamboo internodes. The absence of *Sabethes* larvae in tree holes indicated the tendency of the species found in this study to oviposit in bamboo internodes, even when tree holes were available. Mangudo et al.^9^ noted that although only 5% of the trees sampled in their study had water-holding holes, only a few contained *Sabethes* larvae. These results suggest that (1) there is a low frequency of *Sabethes* oviposition at ground level, probably due to the greater availability of tree holes at the canopy level, which can contribute to the canopy-dwelling behavior of this genus, and (2) the potential ecological plasticity of these species for efficient use of bamboo internodes. As *Sa. albiprivus* is considered a potential yellow fever virus vector and naturally infects individuals in Argentina^11^ and Brazil^12^, the scenario described above highlights the crucial role of bamboo internodes in epidemiological surveys.

Mangudo et al.^9^ reported that of all the tree holes in which *Sa. purpureus* larvae were found in their study, most (86%) were laterally oriented (angular-side entrances on the tree trunk). This corroborates the results of Mattingly^13^, who showed the tendency of *Sabethes* species to oviposit in cryptic entrances. Our results support the findings of these studies, as several *Sabethes* species were collected from study areas where tree-hole breeding sites were less common than bamboo-hole breeding sites. Our findings also showed that the use of artificial holes in bamboo to collect *Sabethes* species was valid in places where adult individuals were captured, but larvae were not. In addition to increasing the number of species collected, this method is an alternative to mosquito field procedures, in which collectors are exposed to pathogens^14^.

## Data Availability

Data-in-article (Table 1).
